# Evaluating intra‐fraction motion and its dosimetric impact in ethos online adaptive radiotherapy for prostate cancer

**DOI:** 10.1002/pro6.70013

**Published:** 2025-08-20

**Authors:** Yoganathan SA, Ahamed Basith, Rajeev Choudary Apsani, Venkada Manickam Gurusamy, Amine Khemissi, Satheesh Paloor, Saju Divakar, Sarah McCabe, Rabih Hammoud, Noora Al‐Hammadi

**Affiliations:** ^1^ Radiation Oncology, Saint John Regional Hospital Horizon Health Network Saint John New Brunswick Canada; ^2^ Radiation oncology Hamad Medical Corporation Doha Qatar

**Keywords:** Ethos treatment planning system, planning target volume, prostate cancer

## Abstract

**Purpose:**

This study evaluated the geometric and dosimetric uncertainties during online adaptive radiotherapy (ART) for prostate cancer.

**Methods:**

Sequential cone beam computed tomography (CBCT) scans from 52 sessions involving 13 patients were analyzed. An ART plan was generated using CBCT1, followed by a verification scan (CBCT2) acquired 13.1 ± 3.0 minutes before treatment delivery. New contours (prostate, seminal vesicles, bladder, rectum, and bowel) were delineated on CBCT2 and transferred to CBCT1 for dose distribution analysis. Three dimensional contour variations were quantified, new planning target volume (PTV) margins were calculated, and their dosimetric benefits were assessed.

**Results:**

No significant differences were observed in the high‐dose volumes (V60 Gy and V57 Gy) of the bladder and rectum (*P* > 0.05). PTV margins were 2.6 mm, 2.4 mm, and 2.6 mm in the lateral, vertical, and longitudinal directions for the prostate, and 3.9 mm, 3.8 mm, and 4.3 mm for the seminal vesicles, respectively. These margin reductions led to a 37% reduction in the dose to the surrounding critical organs, while maintaining consistent target coverage.

**Conclusion:**

This study supports symmetric PTV margins of 3 mm for the prostate and 4.5 mm for the seminal vesicles in online ART, thereby contributing to the development of optimized treatment strategies for prostate cancer.

## Graphical abstract

The figure illustrates their cumulative frequency distributions of motion. The data indicate that movements of the prostate were generally smaller than those of the seminal vesicles. Specifically, the prostate exhibited maximum motion predominantly in the inferior and anterior directions, whereas the seminal vesicles showed maximum motion in the posterior and left directions. This distinction highlights the differing patterns of intrafractional motion between these anatomical structures.

**Figure G1**. Cumulative frequency distribution of intrafractional motion for the prostate.

**Figure G2**. Cumulative frequency distribution of intrafractional motion for the seminal vesicles.

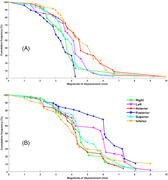

## 1 INTRODUCTION

Variability in internal organ filling and weight loss induces anatomical changes during daily fractions, posing challenges that compromise the precision of conformal dose delivery.^1^, ^2^ Consequently, the original plan, formulated based on planning computed tomography (CT), may prove to be inadequate for accurate administration. Therefore, adaptive treatments have been advocated to address this issue.^3^, ^4^, ^5^

Adaptive radiation therapy (ART) employs innovative strategies to accommodate anatomical changes and ensure precise treatment delivery despite variations. ART is typically classified into two categories: offline and online treatments.^5^ Offline ART involves designing new treatments before therapy initiation, whereas online ART adjusts treatments daily while the patient is under treatment.^5^

Initial studies explored offline ART, with some focusing on rectifying anatomical differences through new plans generated offline on re‐planning CT scans or utilizing plan libraries.^6^ ^,^ ^7^ However, these methods may not completely capture all daily anatomical variations, particularly in complex cases, such as prostate treatments, where the positions of critical structures, such as the prostate, seminal vesicles, bladder, rectum, and bowel, can fluctuate significantly.^5^ Strict adherence to bladder and rectal filling protocols can be challenging for patients, further complicating consistent treatment delivery.

Online ART has emerged as a promising solution by dynamically adjusting treatment plans in response to daily anatomical changes.^3^, ^4^, ^5^ This approach utilizes advanced imaging techniques, such as magnetic resonance imaging (MRI)^8^, ^9^, ^10^, ^11^, ^12^ or cone‐beam CT (CBCT)^13^ ^‐^ ^15^ to facilitate the real‐time adaptation of treatment strategies. The Ethos ART system, developed by Varian Medical Systems, represents a significant advancement in radiotherapy, utilizing CBCT‐based online ART to enhance treatment precision and effectiveness.^14^ These techniques enable clinicians to monitor and adapt to anatomical variations during radiotherapy, ensuring that patients receive optimized treatment while minimizing the impact of daily physiological changes.

However, the online ART involves multiple complex processes, including image acquisition, contouring, plan adaptation, quality assurance, and treatment delivery, which collectively consume considerable time, typically up to 30 minutes.^15^, ^16^, ^17^ This time frame introduces a window during which further anatomical changes can occur, potentially affecting treatment accuracy and efficacy.

Recent studies have primarily focused on evaluating setup errors and planning target volume (PTV) margins for online ART, emphasizing the importance of PTV margins for intrafractional anatomical changes.^17^, ^18^, ^19^ However, a critical gap remains in comprehensively evaluating the dosimetric impact of intrafractional motion on prostate ART, particularly through the use of a second CBCT scan.

Therefore, this study aimed to evaluate the dosimetric uncertainties associated with online prostate ART due to anatomical variations observed on CBCT2 scan. Additionally, we sought to quantify the setup errors to determine the optimal PTV margins for online ART. At our center, the online ART process with Ethos involves generating a treatment plan based on the first CBCT scan (CBCT1), followed by acquiring a subsequent CBCT scan (CBCT2) immediately before beam delivery to verify the patient setup. This study compared the anatomical changes observed between CBCT1 and CBCT2, focusing on their dosimetric implications for prostate ART.

## 2 METHODS AND MATERIALS

### 2.1 Patient selection and study design

This retrospective study was conducted in accordance with our institutional review board‐approved protocol (MRC‐01‐24‐296). Thirteen patients with prostate cancer who received treatment between June 2022 and July 2024 were included. All patients had medium‐/intermediate‐risk prostate cancer and were prescribed a total dose of 60 Gy administered in 20 fractions. All patients underwent planning CT in the supine position using a Siemens CT scanner with a slice thickness of 3 mm.

All patients were instructed to follow a bladder protocol, which involved drinking 90 mL of water over a 15‐minute period. While our goal was to maintain a consistent bladder volume during treatment, patients were advised not to stress about achieving this, as the online ART system would automatically adjust for variations in bladder volume. All CT images were imported into the Eclipse treatment planning system (TPS) (Varian Medical Systems, Palo Alto, CA, USA), where a qualified radiation oncologist delineated the target volumes and organs at risk (OARs). The clinical target volume (CTV) included the prostate with either full or proximal seminal vesicles, depending on the risk. We started our online ART program with a PTV margin similar to that used for image‐guided radiotherapy (IGRT), which was a 7 mm margin all around, except 5 mm in the posterior direction.

All CT images and contours were transferred to Ethos TPS (Varian Medical Systems, Palo Alto, CA, USA) for initial planning. The Ethos TPS uses templates to define clinical goals. During optimization, the clinical goals were reordered depending on their importance. The same clinical goal order was used during on‐couch adaptation. After optimization, the Ethos TPS generated five automated plans: fixed‐field intensity modulated radiotherapy (IMRT) (7, 9, and 12 fields) and two volumetric modulated arc therapy (VMAT). In our cases, the initial treatment plans were created using 12‐field IMRT or 2‐ or 3‐arc VMAT in the Ethos TPS.

### 2.2 Adaptive workflow

**FIGURE 1:**
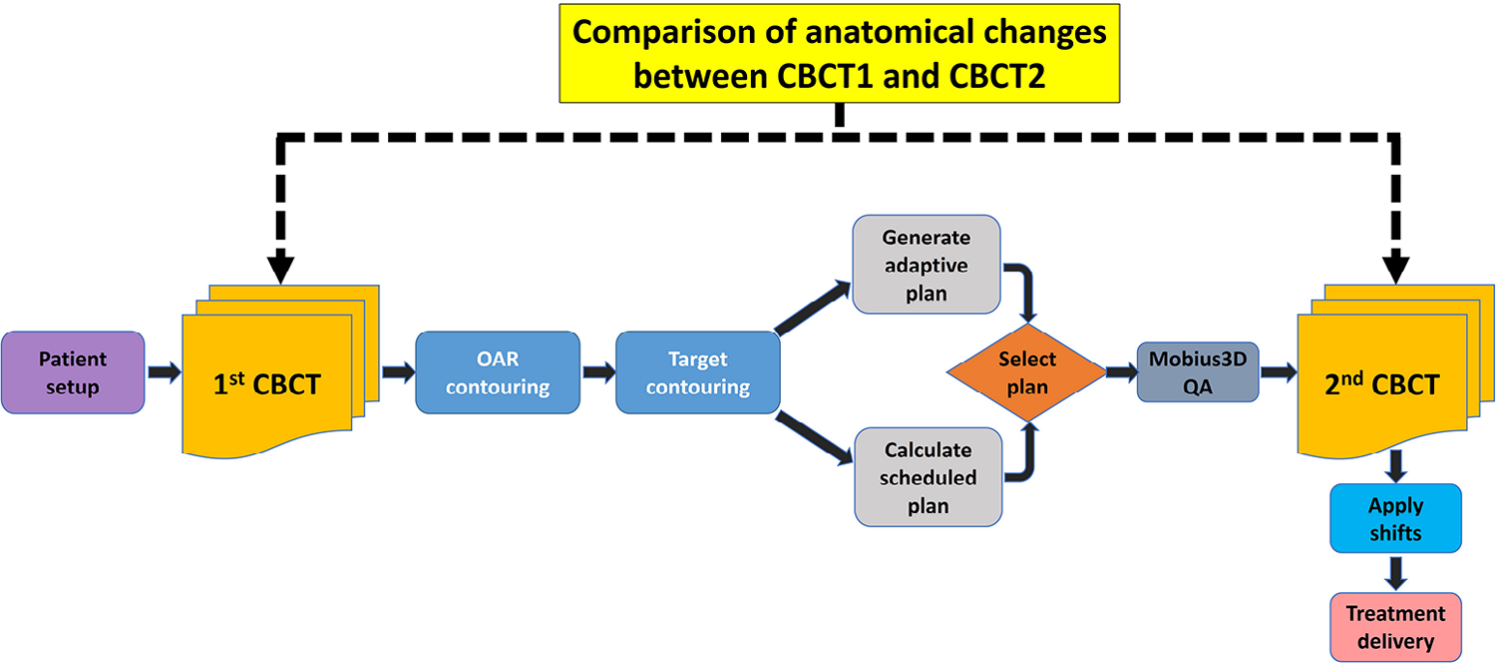
Workflow of the study. CBCT, cone beam computed tomography; OAR, organs‐at‐risk.

The workflow of this study is illustrated in Figure 1. The patients underwent treatment beginning with the acquisition of CBCT1 immediately after the setup. Subsequently, the Ethos system employed deep learning models to automatically generate contours for OARs, including the prostate, seminal vesicles, bladder, rectum, and bowel. A RO reviewed these auto‐contoured structures and potentially adjusted them for accuracy. The CTV was defined by combining the prostate and seminal vesicles. Using the previously defined margins (initial planning stage), the PTV was automatically created for each session, followed by plan generation.

The Ethos adaptive system (version 1.1) uses deformable image registration to fuse planning CT with CBCT1 data, creating a synthetic CT (sCT) for accurate dose calculations. The Ethos system utilized the same beam geometry employed during the initial planning and used the Acuros algorithm for dose calculation. Two distinct plans were generated: a scheduled plan recalculating the original plan based on the current anatomy and an adaptive plan entirely re‐optimized for adaptability. Approval of the adaptive plan required both clinical validation by the RO and technical approval by a medical physicist.

Following plan generation, the adaptive plan was transferred to the Mobius system for verification of the secondary dose calculation. This involved comparing the resultant dose distribution with the original Ethos calculation using the 3%/3 mm gamma evaluation criteria. Before beam delivery, a verification CBCT (CBCT2) scan was performed to verify the final patient position, with the necessary couch shifts (3D) applied as per the protocol.

### 2.3 Evaluation

In this study, the geometric and dosimetric variations observed in the CBCT2 across 52 sessions (one session per week) involving 13 patients were analyzed. The session images, including sCT, CBCT1, CBCT2, and the adaptive plan, were exported from the Ethos TPS and imported into the Eclipse TPS. However, because the registration data for sCT, CBCT1, and CBCT2 were not exported by the Ethos TPS, manual registration was performed in Eclipse. In each session, the RO manually delineated new contours for the prostate, seminal vesicles, bladder, rectum, and bowel by using CBCT2. CBCT2 was registered with CBCT1 using translational shifts to replicate the couch adjustments made during the patient treatment based on CBCT2 matching. The contours from CBCT2 were then transferred to CBCT1. Subsequently, the sCT was registered with CBCT1 using the Digital Imaging and Communications in Medicine origin, and the contours from CBCT1, including those transferred from CBCT2, were transferred to the sCT. This step was crucial for evaluating the dosimetric impact of intrafractional motion, given that the adaptive plan was based on the sCT.

Dose distributions from the sCT were overlaid onto new contours from the CBCT2, and dose‐volume histograms (DVHs) were analyzed. The key parameters assessed included CTV coverage (D97%); bladder and rectum volumes receiving 60 Gy, 50 Gy, and 30 Gy (V60Gy, V50Gy, and V30Gy); bowel D2cc; and penile bulb mean dose (Dmean). To capture deviations more accurately, we compared the absolute volumes of the bladder and rectum rather than the relative volumes, as the latter would not fully account for the variations in the bladder and rectum volumes between CBCT1 and CBCT2.

Additionally, CTV contours from the CBCT1 and CBCT2 were overlaid, and 3D contour differences were calculated for each fraction using MATLAB (MathWorks, Natick, MA, USA). Systematic and random errors were computed in three dimensions to determine the PTV margin using the van Herk formula (2.5Σ+0.7σ),^20^ where Σ represents the systematic error, calculated as the standard deviation of the individual mean set‐up error about the overall population mean, and σ represents the random error, calculated as the mean of the individual random errors.^21^

To assess the dosimetric benefits of the new PTV margin (the online ART margin ‐ 3mm for prostate and 4.5mm for seminal vesicles), we compared it with the standard IGRT margin (7mm margin all around except 5mm to the posterior direction) using data from eight randomly selected sessions across different patients. The new online ART PTV margins were applied to create new PTVs using the CTV (prostate and seminal vesicles) from CBCT1/sCT, and new treatment plans were generated based on these new PTVs using a similar beam geometry and optimization objectives. We then compared the CTV coverage and OAR doses (bladder and rectum) between the IGRT and new online ART margins, specifically transferring CTVs and OARs from CBCT2 to the sCT for comparison. This approach highlights the potential for improved organ sparing using the newly proposed PTV margins.

## 3 RESULTS

**FIGURE 2:**
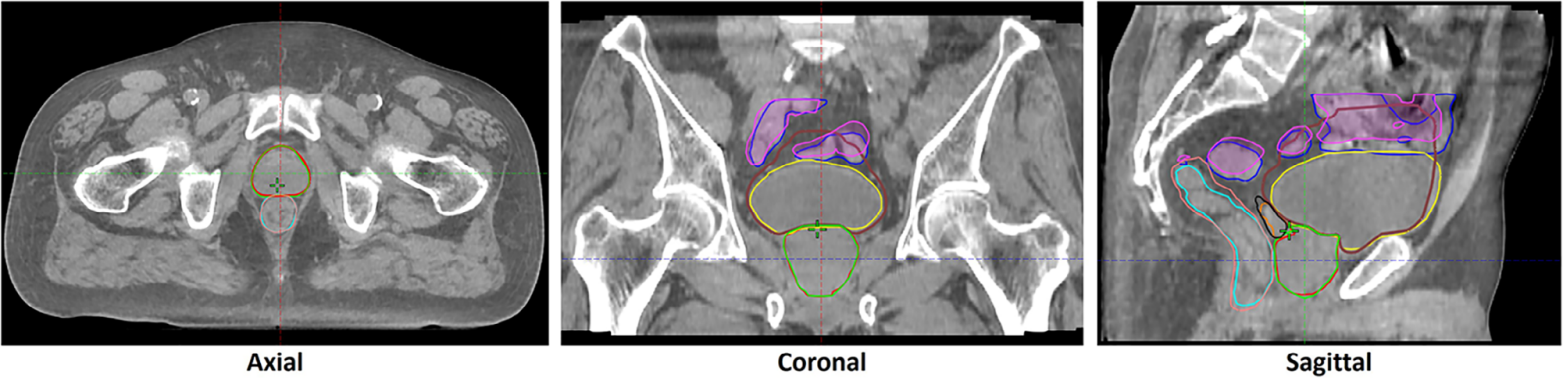
Intra‐fractional variation in contours between two CBCT scans, CBCT1 vs CBCT2. Prostate (red vs green), seminal vesicles (orange vs black), bladder (yellow vs brown), rectum (cyan vs pink), and bowel (blue vs purple). CBCT, cone beam computed tomography.

**FIGURE 3:**
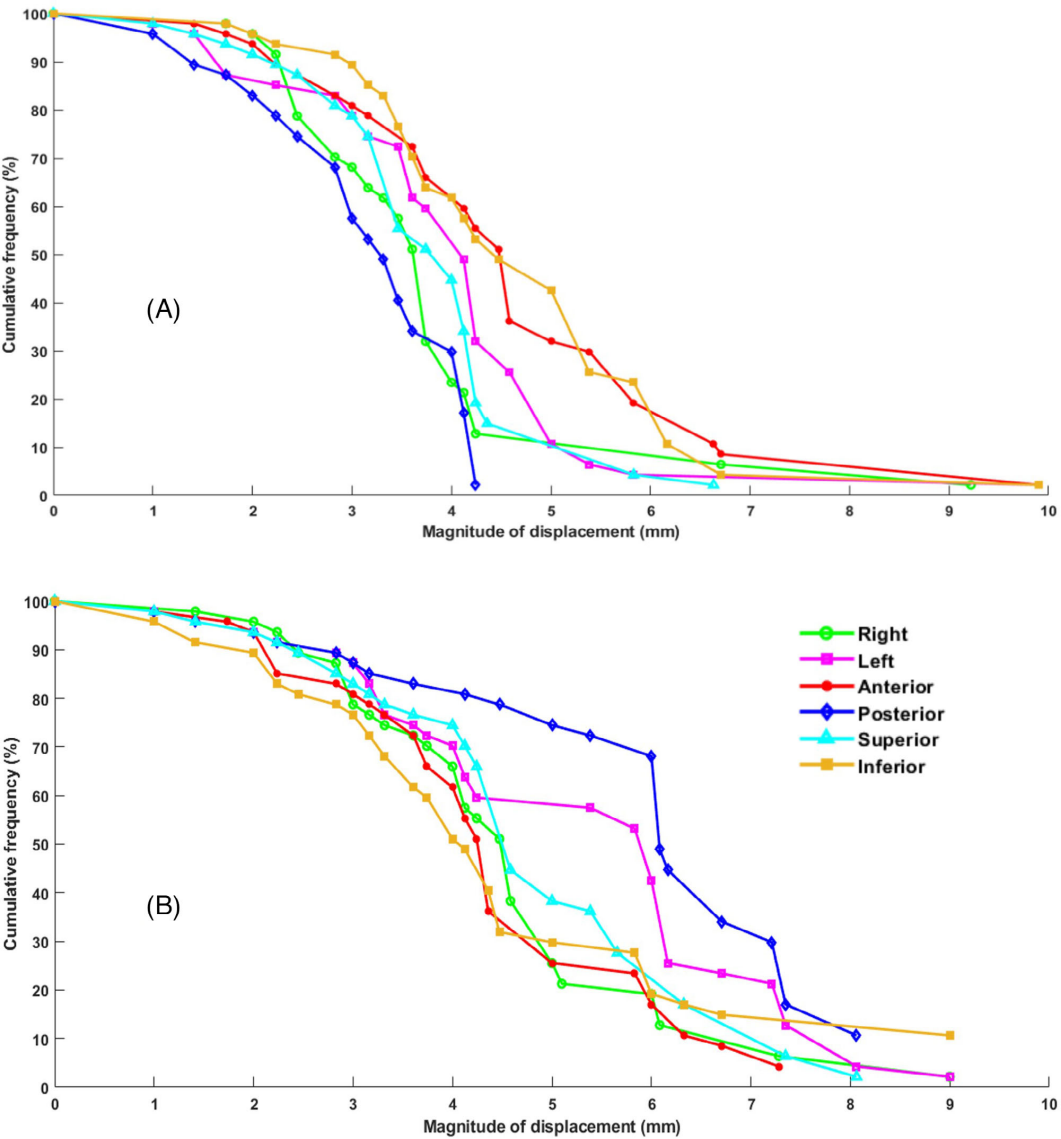
Cumulative frequency distribution of intrafractional motion for the prostate (a) and seminal vesicles (b).

The mean interval between the CBCT1 and CBCT2 scans was 13.1 ± 3 minutes. During this period, notable intrafractional motion was observed, as shown in Figure 2, which illustrates the intrafractional contour changes between CBCT1 and CBCT2. The contour differences were analyzed for both the prostate and seminal vesicles, and Figure 3 illustrates their cumulative frequency distributions of motion. These data indicate that the movements of the prostate were generally smaller than those of the seminal vesicles. Specifically, the prostate exhibited maximum motion predominantly in the inferior and anterior directions, whereas the seminal vesicles exhibited maximum motion in the posterior and left directions. This distinction highlights the different patterns of intrafractional motion between these anatomical structures.

The mean displacements of the prostate between CBCT1 and CBCT2 were 3.8 ± 1.4 mm, 3.8 ± 1.5 mm, and 3.9 ± 1.5 mm in the lateral, vertical, and longitudinal directions, respectively. For the seminal vesicles, these displacements were 4.2 ± 1.8 mm, 4.2 ± 1.7 mm, and 4.2 ± 1.9 mm, respectively. Random errors were calculated as 1.2 mm, 1.3 mm, and 1.3 mm for the prostate and 1.5 mm, 1.4 mm, and 1.5 mm for the seminal vesicles, respectively, whereas systematic errors were 0.7 mm, 0.6 mm, and 0.7 mm for the prostate and 1.1 mm, 1.1 mm, and 1.3 mm for the seminal vesicles, respectively.

To ensure a 95% dose coverage for 90% of the sessions, the margins were calculated using the van Herk formula. The resulting PTV margins were 2.6 mm, 2.4 mm, and 2.6 mm for the prostate and 3.9 mm, 3.8 mm, and 4.3 mm for the seminal vesicles in the lateral, vertical, and longitudinal directions, respectively.

**FIGURE 4:**
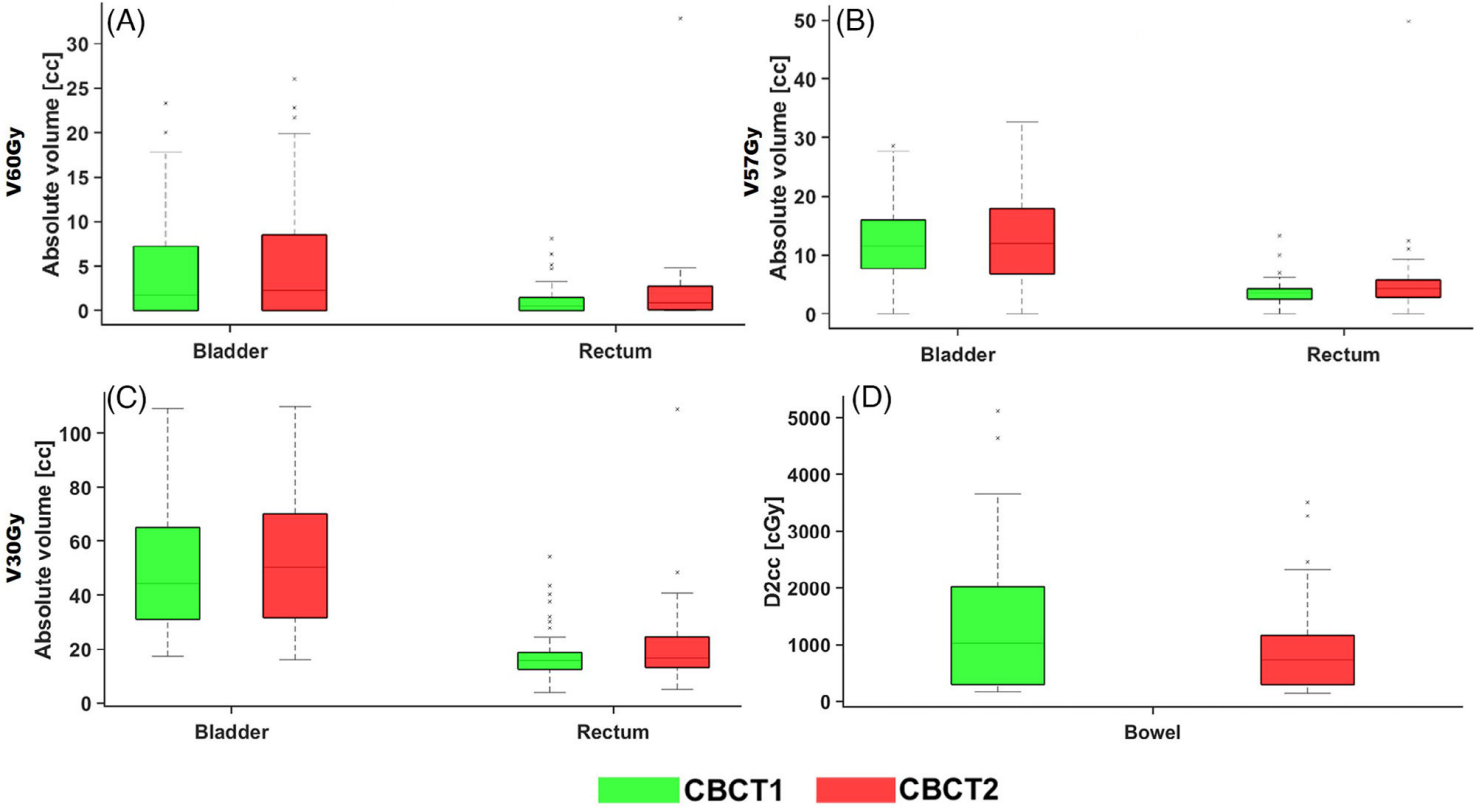
OAR dose differences between CBCT1 and CBCT2 scans for the bladder and rectum: (a) V60Gy (cc), (b) V57Gy (cc), (c) V30Gy (cc), and for the bowel: (d) D2cc (cGy). OAR, organs‐at‐risk; CBCT, cone beam computed tomography.

Significant changes were observed in bladder volume, increasing by 27% (mean: 215 ± 113 cc vs 260 ± 120 cc) between the CBCT1 and CBCT2 scans, whereas the volumes of the CTV and rectum remained stable (<6%). No statistically significant differences were observed in CTV coverage (D97%) between CBCT1 and CBCT2 (*P* > 0.05). Figure 4 shows the differences in OAR doses between CBCT1 and CBCT2. Although the DVH parameters for the bladder were slightly higher in the CBCT2 results, these differences were not statistically significant (*P* > 0.05). Conversely, rectal doses at V30 were significantly higher in the CBCT2 (mean: 17.9 ± 9.7 cc vs. 21.3 ± 15.6 cc, *P* < 0.035), whereas bowel doses (D2cc) were significantly lower (mean: 13.3 ± 11.9 Gy vs 9.1 ± 8.1 Gy, *P* < 0.0001).

Table 1 compares the DVH differences between the standard IGRT and online ART margins for the CTVs, rectum, and bladder. The results showed that the new PTV margin improved sparing of OARs, with an average dose reduction of approximately 37% compared with the IGRT margin. Additionally, the online ART margin effectively accounted for the intrafractional variations in the CTVs between CBCT1 and CBCT2, as evidenced by the similar CTV dose coverage between the IGRT and OART margins.

**TABLE 1:** Comparison of differences in DVH between the IGRT and OART margins

Structures	DVH parameter	IGRT‐margin	OART‐margin
CTV	D98% (cGy)	5910.5 ± 63.8	5903.3 ± 76.3
Rectum	V57Gy (cc)	3.0 ± 1.2	1.5 ± 1.1
	V30Gy (cc)	14.2 ± 2.5	11.4 ± 3.7
Bladder	V57Gy (cc)	11.8 ± 4.5	6.3 ± 1.6
	V30Gy (cc)	37.8 ± 13.2	26.3 ± 9.8

Abbreviations: CTV, clinical target volume; DVH, dose‐volume histograms; IGRT, image‐guided radiotherapy; OART, online adaptive radiotherapy.

## 4 DISCUSSION

In this study, we investigated intrafractional motion and its implications in online ART for prostate cancer. Previous studies have largely addressed the PTV margin of the prostate in CBCT‐based online ART. To the best of our knowledge, this study is the first to comprehensively evaluate PTV margins and analyze the dosimetric impact of intrafractional motion in online prostate ART.

We observed that CTV coverage remained unchanged, largely because of the initially larger IGRT margins. We observed minimal dosimetric differences in the rectal and bladder volumes between the CBCT1 and CBCT2 scans. However, significant changes were observed in the low‐dose regions of the rectum (V30 Gy), with reduced bowel doses noted on the CBCT2 scan. These variations were attributed to the increased bladder volumes between scans, causing superior displacement of the bowel, thereby decreasing its dose. We also noted challenges in using relative volume constraints for comparing CBCT scans. For instance, despite both the CBCT1 and CBCT2 scans showing a V60 value of 3%, the larger bladder volume in the CBCT2 scan resulted in different absolute volumes. Consequently, we adjusted our approach to use absolute volumes for DVH comparisons between the bladder and rectum.

In the evaluations, all patients adhered to the conventional prostate IGRT margins (5 mm posteriorly and 7 mm overall). However, the study indicated an opportunity for reduction. Specifically, our findings suggest that symmetrical PTV margins of 3 mm for the prostate and 4.5 mm for the seminal vesicles are adequate. Despite performing couch shifts based on CBCT2 data, residual errors persisted, possibly due to limitations in the 3D couch adjustments of the Ethos system or complex deformation of the prostate and seminal vesicles, which may not be fully corrected by couch shifts alone. Therefore, smaller margins are warranted, as indicated in our study. These reduced margins offer the potential to lower the dose to surrounding organs, such as the rectum and bladder. This was further supported by a dosimetric comparison between conventional IGRT margins and our new online ART margin treatment plans, which showed that the new PTV margin resulted in a 37% reduction in OAR doses while maintaining the same CTV coverage.

Our online ART process, averaging 13.1 ± 3.0 minutes (excluding delivery time), is consistent with other studies employing the Ethos system for prostate treatments. Typically, Ethos online ART sessions are completed within a narrow window of 25–30 minutes (including delivery time), in contrast to MRI‐guided online ART, which often exceeds 30 minutes.^15^, ^16^, ^17^ The treatment duration significantly influences prostate motion, suggesting potential advantages in concluding treatment within a shorter timeframe. VMAT techniques were predominantly used because of their superior plan quality, particularly in reducing low‐dose streaks, compared with IMRT setups. Although VMAT plan generation during adaptive sessions in Ethos typically required 9–10 minutes, versus 2–3 minutes for IMRT, the delivery time for VMAT was shorter (approximately 9 minutes compared to 14 minutes for 12‐field IMRT), resulting in comparable overall treatment durations.

A significant challenge we encountered was the time‐intensive contouring and planning process inherent to online ART, which sometimes led to variations in bladder filling between the initial CBCT1 and pre‐treatment CBCT2 scans. Despite these variations, our clinical focus remained on achieving precise target alignment for optimal coverage of the prostate and proximal seminal vesicles. Notably, bladder volume changes primarily occurred at the superior aspect, typically outside the treatment field, minimizing the dosimetric impact.

Our findings on the PTV margins in prostate radiotherapy align with those of previous studies (Table 2). Morgan et al.^18^ evaluated PTV margins for postoperative prostate radiation therapy between CBCT1 and CBCT2, reporting margins of 1.6 mm, 2.0 mm, and 2.2 mm (lateral, vertical, and longitudinal axes, respectively). Byrne et al.^19^ assessed the PTV margins for prostate online ART using the Ethos system and found that margins of 4.0 mm and 5.0 mm for the prostate and seminal vesicles, respectively, provided sufficient target coverage. In a study using magnetic resonance‐based online ART, Keizer et al. concluded that a 5.0 mm margin was adequate. These studies indicate that PTV margins can vary based on the treatment duration, imaging frequency, and specific workflow. Morgan et al.^18^ and Byrne et al.^19^ found that shorter treatment times (approximately 10–17 minutes) allowed smaller margins (1.6–4.0 mm) to maintain precise target coverage. Conversely, Keizer et al.^17^ observed longer treatment durations (approximately 33 min), which necessitated slightly larger margins (approximately 5.0 mm) owing to potential intrafractional motion and setup uncertainties. Our findings (approximately 13.1 ± 3 minutes) closely align with those of Morgan et al., suggesting that margins of approximately 3.0 mm for the prostate and 4.5 mm for the seminal vesicles are adequate under these circumstances.

**TABLE 2:** Comparison between planning target volume margins observed in the current study and previous studies.

Study	Details	Duration (minutes)	Structure	Right‐Left (mm)	Anterior‐Posterior (mm)	Superior‐Inferior (mm)
Byrne et al.^19^	CBCT‐based OART with verification imaging before delivery	17.1 ± 5.8	Prostate	4.0		
			Seminal vesicles	5.0		
Keizer et al.^17^	MRI based OART	33.1 ± 4.7	Prostate	5.0		
Morgan et al.^18^	Non‐OART, CBCTs from C‐arm linac	10.7 ± 3.0	Clinical target volume	1.6	2.0	2.2
Current study	CBCT‐based OART with verification imaging before delivery	13.1 ± 3.0	Prostate	2.6	2.4	2.6
			Seminal vesicles	3.9	3.8	4.3

Abbreviations: CBCT, cone beam computed tomography; MRI, magnetic resonance imaging; OART, online adaptive radiotherapy.

A significant limitation of our study is the sample size, which included 52 session images. Increasing the number of patients would improve statistical power and broaden the applicability of our findings, prompting further exploration in larger cohorts. Ideally, PTV margins should be evaluated using CBCT images acquired after treatment to account for complete prostate motion. However, our workflow entailed obtaining CBCT scans immediately before “beam‐on” to facilitate real‐time adjustments, thereby enhancing treatment precision. Alternatively, a third CBCT scan could have been considered, although this approach might raise concerns regarding the imaging dose.

## 5 CONCLUSION

In this study, we assessed the intrafractional movements of the prostate and seminal vesicles, along with their dosimetric impact, using sequential CBCT scans during online ART. The high‐dose volumes (V60Gy and V57 Gy) for the bladder and rectum showed no significant differences (*P* > 0.08). We found that the PTV margins could be reduced to 2.6 mm, 2.4 mm, and 2.6 mm for the prostate and 3.9 mm, 3.8 mm, and 4.3 mm for the seminal vesicles in the lateral, vertical, and longitudinal directions, respectively. These reductions in the PTV margins could lower the doses to the surrounding OARs by 37% while ensuring consistent coverage of the CTV. These results provide valuable insights for optimizing online adaptive radiotherapy strategies for prostate cancer treatment.

## CONFLICT OF INTEREST STATEMENT

The authors declare that they have no competing interests.

## ETHICAL APPROVAL

This retrospective study was conducted in accordance with our institutional review board‐approved protocol (MRC‐01‐24‐296), which waived the requirement for written informed consent

## Data Availability

Datasets and other files generated, analyzed, or processed in this study are available upon request from the corresponding author.
